# 5-Aminolevulinic acid fermentation using engineered *Saccharomyces cerevisiae*

**DOI:** 10.1186/s12934-019-1242-6

**Published:** 2019-11-07

**Authors:** Kiyotaka Y. Hara, Masaru Saito, Hiroko Kato, Kana Morikawa, Hiroshi Kikukawa, Hironari Nomura, Takanori Fujimoto, Yoko Hirono-Hara, Shigeyuki Watanabe, Kengo Kanamaru, Akihiko Kondo

**Affiliations:** 1 Department of Environmental and Life Sciences, School of Food and Nutritional Sciences, University of Shizuoka, 52-1 Yada, Suruga, Shizuoka 422-8526 Japan; 2 Organization of Advanced Science and Technology, Kobe University, 1-1 Rokkodai-cho, Nada-ku, Kobe, 657-8501 Japan; 3 Cosmo Oil Co., Ltd., 1-1-1 Shibaura, Minato-ku, Tokyo, 105-8528 Japan; 4 Department of Chemical Science and Engineering, Graduate School of Engineering, Kobe University, 1-1 Rokkodaicho, Nada-ku, Kobe, 657-8501 Japan; 5 Department of Applied Chemistry in Bioscience, Graduate School of Agricultural Science, Kobe University, Kobe, 657-8501 Japan; 6 Graduate School of Science, Technology and Innovation, Kobe University, 1-1 Rokkodai-cho, Nada-ku, Kobe, 657-8501 Japan; 7 Engineering Biology Research Center, Kobe University, 1-1 Rokkodai-cho, Nada-ku, Kobe, 657-8501 Japan

**Keywords:** 5′-Aminolevulinic acid, Yeast, *Saccharomyces cerevisiae*, Metabolic engineering, Cell factory

## Abstract

**Background:**

5′-Aminolevulinic acid (ALA) is widely used in the pharmaceutical industry, healthcare, and food production, and is a substrate for the biosynthesis of heme, which is required for respiration and photosynthesis. Enhancement of ALA biosynthesis has never been developed in *Saccharomyces cerevisiae*, which is a well-known model microorganism used for bioproduction of many value-added compounds.

**Results:**

We demonstrated that metabolic engineering significantly improved ALA production in *S. cerevisiae*. First, we found that overexpression of *HEM1*, which encodes ALA synthetase, increased ALA production. Furthermore, addition of an optimal amount of glycine, a substrate for ALA biosynthesis, or levulinic acid, an inhibitor of ALA dehydrogenase, effectively increased ALA production. Next, we developed an assay for multiple metabolites including ALA and found that aconitase, encoded by *ACO1* and *ACO2*, is the rate-limiting enzyme of ALA biosynthesis when sufficient glycine is supplied. Overexpression of *ACO2* further enhanced ALA production in *S. cerevisiae* overexpressing *HEM1*.

**Conclusions:**

In this study, ALA production in *S. cerevisiae* was enhanced by metabolic engineering. This study also shows a strategy to identify the rate-limiting step of a target synthetic pathway by assay for multiple metabolites alongside the target product. This strategy can be applied to improve production of other valuable products in the well-studied and well-industrialized microorganism *S. cerevisiae*.
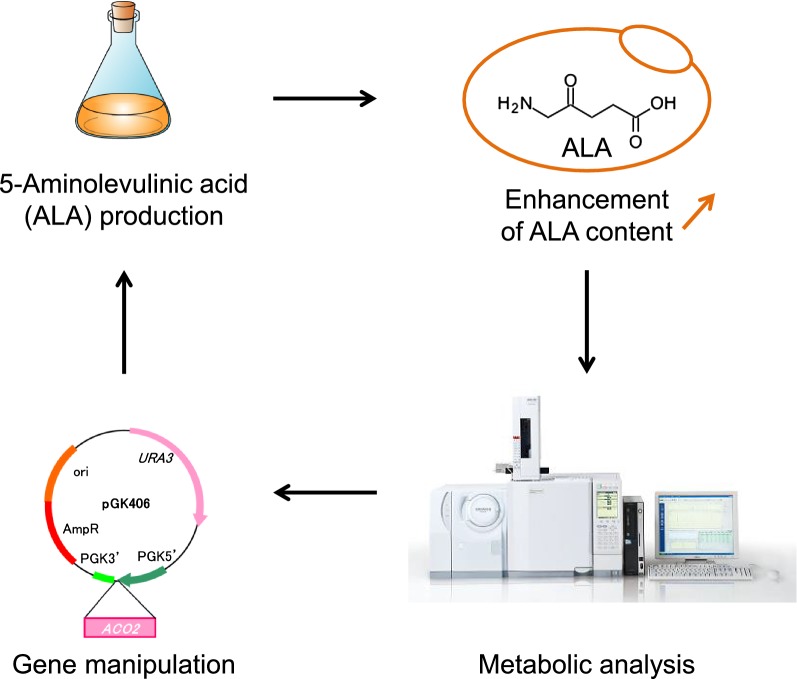

## Background

5′-Aminolevulinic acid (ALA) is an industrial fine chemical, and has important physiological functions in humans and other organisms, including acting as a substrate for heme biosynthesis [1–5]. ALA has been widely used not only as a human healthcare product, but also as an additive in food and fertilizer. In recent years, demand for ALA has increased [2, 4].

ALA biosynthesis occurs via two routes. One is the C5 pathway, which involves three enzymatic reactions and is found in most eubacteria, and in all archaebacteria, algae and plants [2]. In the C5 pathway, tRNA for l-glutamic acid is glutamylated by glutamyl-tRNA synthetase and the resultant l-glutamyl-tRNA(Glu) is then converted to l-glutamate 1-semialdehyde by glutamyl-tRNA reductase. Finally, l-glutamate 1-semialdehyde is converted to ALA, catalyzed by glutamate-1-semialdehyde aminotransferase. The other route to synthesize ALA is the C4 pathway (Shemin pathway) found in animals and fungi. ALA production in the C4 pathway in *Corynebacterium glutamicum* has been improved through metabolic engineering [4]. The C4 pathway depends on the condensation of succinyl-coenzyme A (CoA) and glycine by ALA synthetase (EC 2.3.1.37) [5]. At present, *Rhodobacter sphaeroides* is industrially used to produce ALA via the C4 pathway [5]. However, *R. sphaeroides* cannot be improved through metabolic engineering or synthetic bioengineering.

*Saccharomyces cerevisiae* is a well-studied microorganism, and genetic and metabolic information on it are abundant. *S. cerevisiae* is also “generally recognized as safe”, and commonly used for production of fine chemicals, such as healthcare supplements, food additives, functional feeds, and supplemental fertilizers [6]. An additional advantage of *S. cerevisiae* is tolerance of acidic conditions, enabling production of products such as amino and organic acids. The cellular ability for fine chemical production in *S. cerevisiae* can be enhanced by random mutagenesis, metabolic engineering, and recently, synthetic bioengineering [7]. However, ALA production in *S. cerevisiae* has never been assessed, although there is potential for its improvement through metabolic engineering based on the abundant genetic information and manipulation tools available for this organism.

**Fig. 1:**
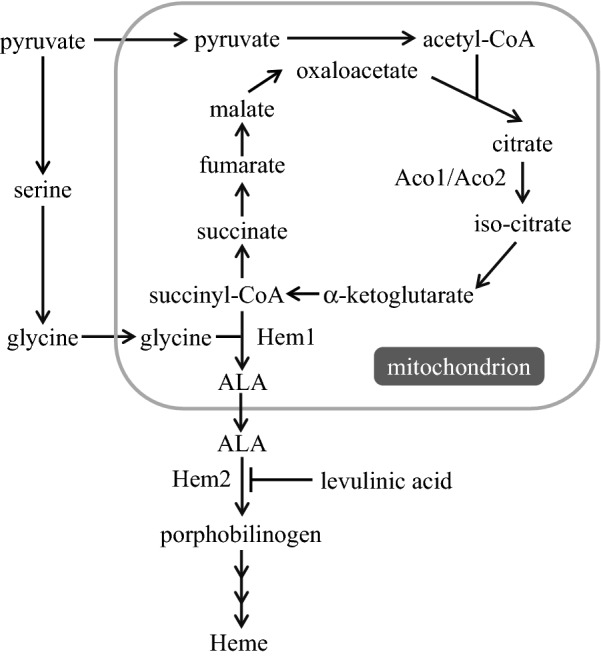
Schematic illustration of the metabolic pathway of 5′-aminolevulinic acid (ALA) synthesis in *Saccharomyces cerevisiae*

In this study, we demonstrated that overexpression of *HEM1* encoding ALA synthetase in *S. cerevisiae* improved ALA production (Fig. 1). Furthermore, we elucidated that overexpression of *ACO2*, which encodes aconitase—the rate-limiting enzyme of ALA biosynthesis when sufficient glycine is available—was effective to enhance ALA production in the *HEM1*-overexpressing *S. cerevisiae*.

## Results

### ALA production in *S. cerevisiae*

**Fig. 2:**
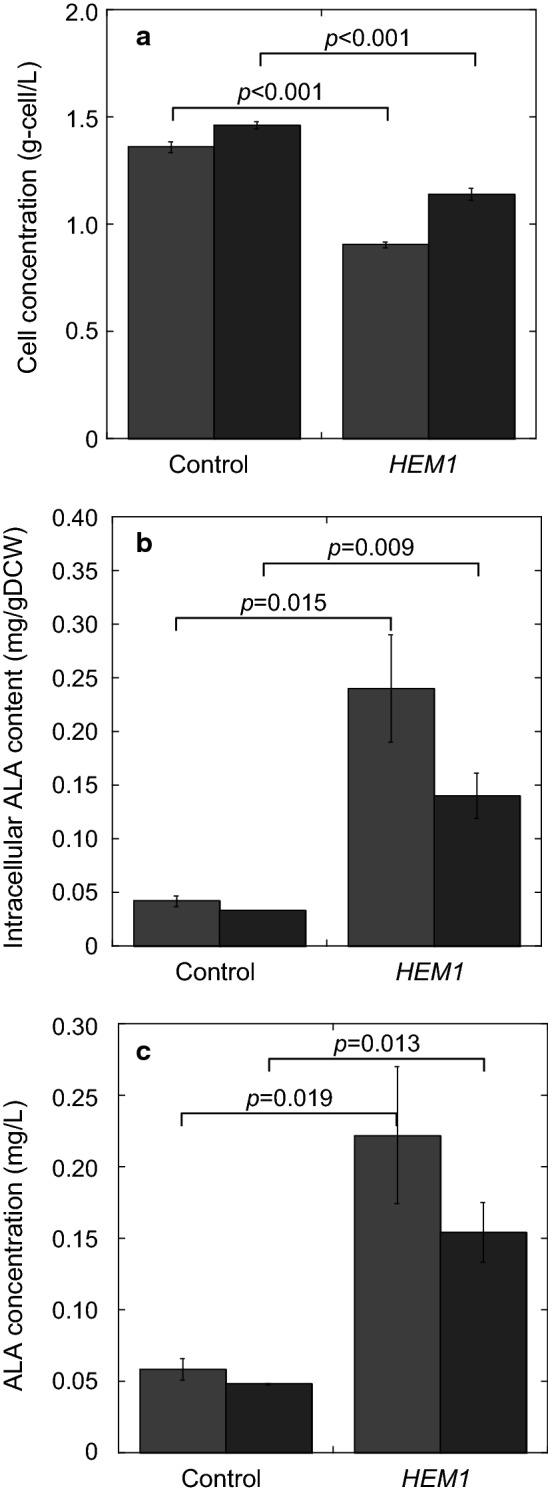
ALA production by the *S. cerevisiae HEM1*-overexpressing strain and the control strain. **a** Cell concentration (g-cell/L); **b** intracellular ALA content (mg/g dry cell weight [DCW]); **c** volumetric ALA concentration (mg/L). Gray and black bars represent values after cultivation for 24 and 48 h, respectively. The values are presented as means, with error bars showing SD (*n *> 3). *p*-values are represented when they show statistically significant differences among the engineered strains by Student’s *t* test (*p* < 0.05, analysis of variance)

Because ALA production using yeast has never been reported, we first evaluated the time course of intracellular ALA content during cell growth of *S. cerevisiae*. As shown in Fig. 2, cell concentration of a vector-control *S. cerevisiae* strain after cultivation for 24 h was almost the same as that after cultivation for 48 h. Intracellular ALA content in this strain after cultivation for 24 h was almost the same as that after cultivation for 48 h. Cellular ALA content is usually measured using high-performance liquid chromatography (HPLC) [4]. However, to both measure ALA content and identify the rate-limiting step of ALA synthesis in *S. cerevisiae*, an assay is required to measure the amount of ALA in combination with other main metabolites. Thus, we developed a new method to measure ALA content in *S. cerevisiae* using gas chromatography-mass spectrometry (GC–MS) in place of conventional HPLC. To detect ALA alongside multiple other main metabolites in *S. cerevisiae*, a new ion monitoring channel was added to the GC–MS multiple metabolite detection assay we previously developed [8] (see “Methods” section for details).

### ALA production using HEM1-overexpressing *S. cerevisiae*

To enhance ALA production, *HEM1*—which encodes ALA synthetase, an enzyme involved in the heme assimilation system in *S. cerevisiae* (Fig. 1)—was overexpressed. ALA fermentation was performed using a vector control strain and a *HEM1*-overexpressing strain. We compared their cell concentrations (growth), intracellular ALA content, and volumetric ALA concentrations after 24 and 48 h of cultivation (Fig. 2). As shown in Fig. 2a, the cell concentration of the *HEM1*-overexpressing strain (0.90 and 1.14 g-cell/L at 24 and 48 h, respectively) was 0.66 and 0.78-fold that of the control strain (1.36 and 1.46 g-cell/L). However, as shown in Fig. 2b, the intracellular ALA content of the *HEM1*-overexpressing strain (0.24 and 0.14 mg/g dry cell weight [DCW]) was 5.7- and 4.2-fold that in the control strain (0.042 and 0.033 mg/g DCW) at 24 and 48 h, respectively. In the *HEM1*-overexpressing strain, considering the total effect of the decrease in the cell concentration (Fig. 2a) and the increase in the intracellular ALA content (Fig. 2b), the volumetric ALA concentration was increased during fermentation (Fig. 2c): the volumetric ALA concentration for the *HEM1*-overexpressing strain (0.22 and 0.15 mg/L at 24 and 48 h, respectively) was 3.8- and 3.2-fold that for the control strain (0.058 and 0.048 mg/L, respectively).

### Effect of levulinic acid addition on ALA production by *S. cerevisiae*

**Fig. 3:**
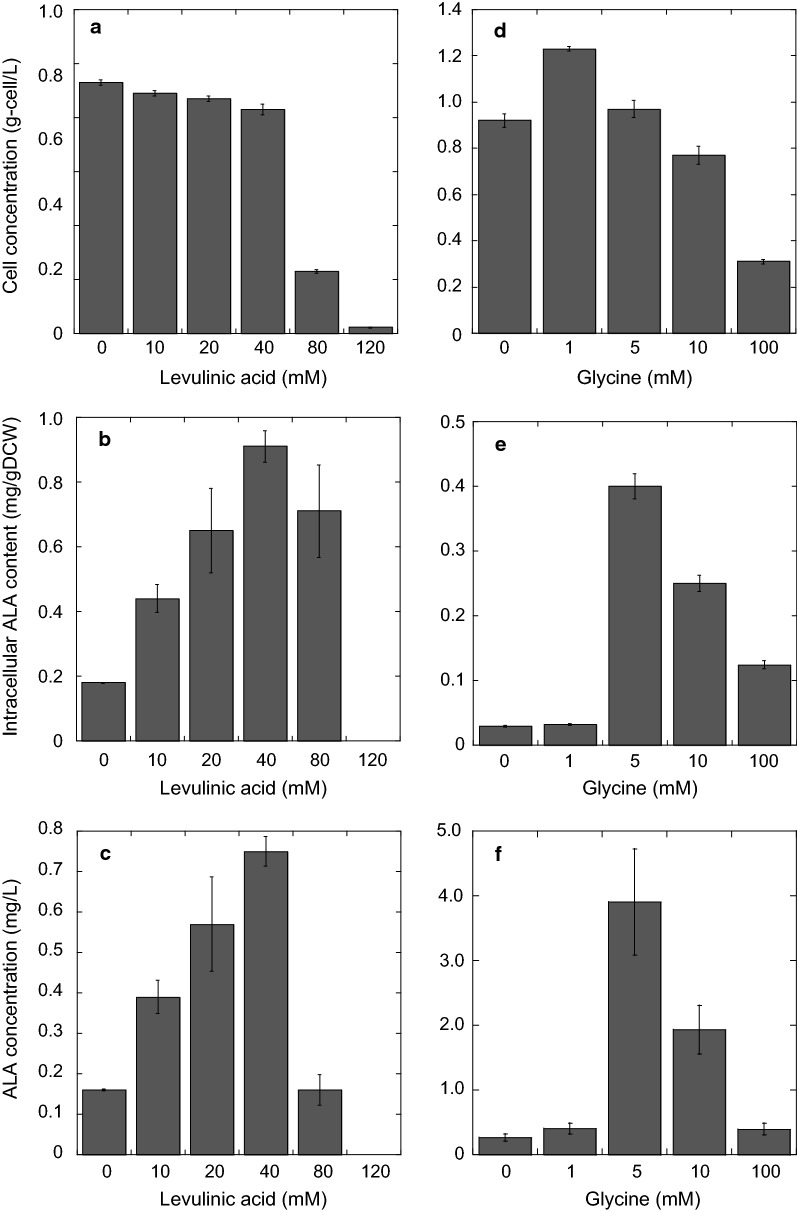
ALA production by the *HEM1*-overexpressing strain with added levulinic acid (**a–c**) or glycine (**d–f**), and cell concentration (g-cell/L) after cultivation for 24 h; **a, d** cell concentration (g-cell/L), **b, e** intracellular ALA content (mg/g DCW); **c, f** volumetric ALA concentration (mg/L). The values are presented as means, with error bars showing SD (*n *= 3)

Levulinic acid is an inhibitor of ALA dehydrogenase (EC 4.2.1.24), encoded by *HEM2* in *S. cerevisiae*, which catalyzes porphobilinogen biosynthesis from ALA [12, 13] (Fig. 1). To enhance ALA production in *S. cerevisiae*, levulinic acid was added to culture of the *HEM1*-overexpressing *S. cerevisiae* strain. We compared cell concentrations, intracellular ALA content, and volumetric ALA concentrations after cultivation for 24 h (Fig. 3). As shown in Fig. 3a, the cell concentration of the *HEM1*-overexpressing strain decreased depending on the levulinic acid concentration. In particular, the addition of more than 80 mM levulinic acid caused a drastic decrease in cell growth. The intracellular ALA content of the *HEM1*-overexpressing strain increased at 0–40 mM added levulinic acid and decreased at 40–120 mM added levulinic acid (Fig. 3b). Considering the cell growth (Fig. 3a) and intracellular ALA content (Fig. 3b), the volumetric ALA concentration was maximized in the *HEM1*-overexpressing strain at 40 mM added levulinic acid (Fig. 3c): the volumetric ALA concentration for the *HEM1*-overexpressing strain after cultivation for 24 h with 40 mM levulinic acid was up to 4.7-fold that with no levulinic acid addition.

### Effect of glycine addition on ALA production in *S. cerevisiae*

Glycine is a substrate for ALA biosynthesis, and glycine addition has been shown to enhance ALA production in *C. glutamicum* [4]. To enhance ALA production in *S. cerevisiae*, glycine was added to culture of the *HEM1*-overexpressing strain in the absence of levulinic acid. We compared cell concentrations (growth), intracellular ALA content, and volumetric ALA concentrations (Fig. 3). As shown in Fig. 3a, the cell concentration of the *HEM1*-overexpressing strain was increased by addition of 1 mM glycine, and decreased by > 10 mM glycine. The intracellular ALA content of the *HEM1*-overexpressing strain was increased by addition of 0–5 mM glycine, and decreased by 5–100 mM added glycine (Fig. 3b). Considering the changes in cell concentration (Fig. 3a) and the intracellular ALA content (Fig. 3b), the volumetric ALA concentration was maximized by addition of 5 mM glycine to culture of the *HEM1*-overexpressing *S. cerevisiae* strain (Fig. 3c). The maximized volumetric ALA concentration produced by the *HEM1*-overexpressing strain after cultivation for 24 h with 5 mM glycine addition reached up to 14.4-fold that with no glycine addition.

### Additional overexpression of ACO1 and ACO2 in HEM1-overexpressing *S. cerevisiae* in the presence of glycine

From the metabolic assay using GC–MS, we determined that the content of citrate and isocitrate (which could not be distinguished from each other by our method) decreased with the increase in ALA production on addition of glycine to the *HEM1*-overexpressing strain (Table 1). This finding indicates that glycine supplementation to the *HEM1*-overexpressing strain stimulates ALA biosynthesis, and also enhances carbon flow to succinyl-CoA (Fig. 1).

**Table 1:** Effect of glycine addition to *HEM1*-overexpressing *Saccharomyces cerevisiae* culture on intracellular citrate and isocitrate levels

Added glycine concentration (mM)	Citrate + isocitrate content (%)	Glycine content (%)
0	1.28	0.025
2.5	0.88	0.071
5.0	0.39	1.40

Samples were taken after culture for 24 h

The culture conditions are described in “Methods”

To elucidate whether there was a shortage of isocitrate in the *HEM1*-overexpressing strain when glycine was supplied, we overexpressed Aco1 and Aco2 in this strain (see Fig. 1). *ACO1* and *ACO2* are annotated as encoding aconitase [9] and putative mitochondrial aconitase isozyme [10], respectively, in the *Saccharomyces* Genome Database (https://www.yeastgenome.org/). Their overexpression was confirmed by measuring the relative mRNA levels of the *ACO1* and *ACO2* genes by reverse transcription quantitative PCR (RT-qPCR) (Additional file 1: Figure S1).

The intracellular ALA content in the *HEM1* single-overexpression strain and that in the *HEM1*/*ACO1* and *HEM1*/*ACO2* double-overexpression strains were compared using the metabolic assay we developed. We found that the intracellular ALA content in the *HEM1*/*ACO1* double-overexpression strain was almost the same as that in the *HEM1* single-overexpression strain. However, the intracellular ALA content in the *HEM1*/*ACO2* double-overexpression strain (1.38 and 1.29 mg/g DCW) was 3.7- and 4.4-fold that in the *HEM1* single-overexpression strain at 24 and 48 h, respectively. As a result of the equal cell growth but increased intracellular ALA content in the *HEM1/ACO2* double-overexpression strain when glycine was added to the culture, the volumetric ALA concentration in this strain (1.31 and 1.36 mg/L) was 3.7- and 4.4-fold that in the *HEM1* single-overexpression strain at 24 and 48 h, respectively.

## Discussion

In this study, we improved ALA production in *S. cerevisiae* through metabolic engineering of the heme biosynthesis pathway (Fig. 1). Overexpression of *HEM1*, encoding ALA synthetase, increased ALA production more than threefold (Fig. 2c), although expression of this gene resulted in decreased cell growth (Fig. 2a). The reason the intracellular ALA content after cultivation for 48 h was lower than that after 24 h (Fig. 2b) was because of the metabolism of ALA into heme and dimerization of ALA. The increase in ALA biosynthesis by overexpression of ALA synthetase was similar to that observed in *C. glutamicum* [4]. *HEM2* encodes 5-aminolevulinate dehydratase, which catalyzes the conversion of ALA to porphobilinogen in heme biosynthesis. Thus, it is expected that deletion of *HEM2* would further enhance ALA biosynthesis. However, in our study, such clones did not grow after the introduction of a PCR fragment to delete *HEM2*. This result indicates that porphobilinogen synthesis is critical to supply the heme that is essential for cell growth of *S. cerevisiae*. Previously, in *Escherichia coli*, direct engineering of the *hemB* open reading frame (corresponding to *HEM2* in *S. cerevisiae*) to inhibit ALA dehydrogenase activity increased ALA accumulation [11]. Therefore, we tested the addition of levulinic acid, an inhibitor of ALA dehydrogenase, to culture of *HEM1*-expressing *S. cerevisiae* [12, 13]; as expected, levulinic acid addition enhanced ALA production (Fig. 3a–c). This result indicates the possibility to enhance ALA production through intracellular biosynthesis of levulinic acid by metabolic engineering.

In *R. sphaeroides* and *C. glutamicum*, glycine is critical in enhancing ALA production because ALA synthetase Hem1 converts it to ALA. Indeed, here, the addition of 5 mM glycine resulted in 4.0 mg/L ALA production in the *HEM1* overexpressing *S. cerevisiae* (Fig. 3f), although a high concentration of glycine inhibited the growth of this strain (Fig. 3d). To improve ALA production, further metabolic engineering of *S. cerevisiae* is necessary.

**Fig. 4:**
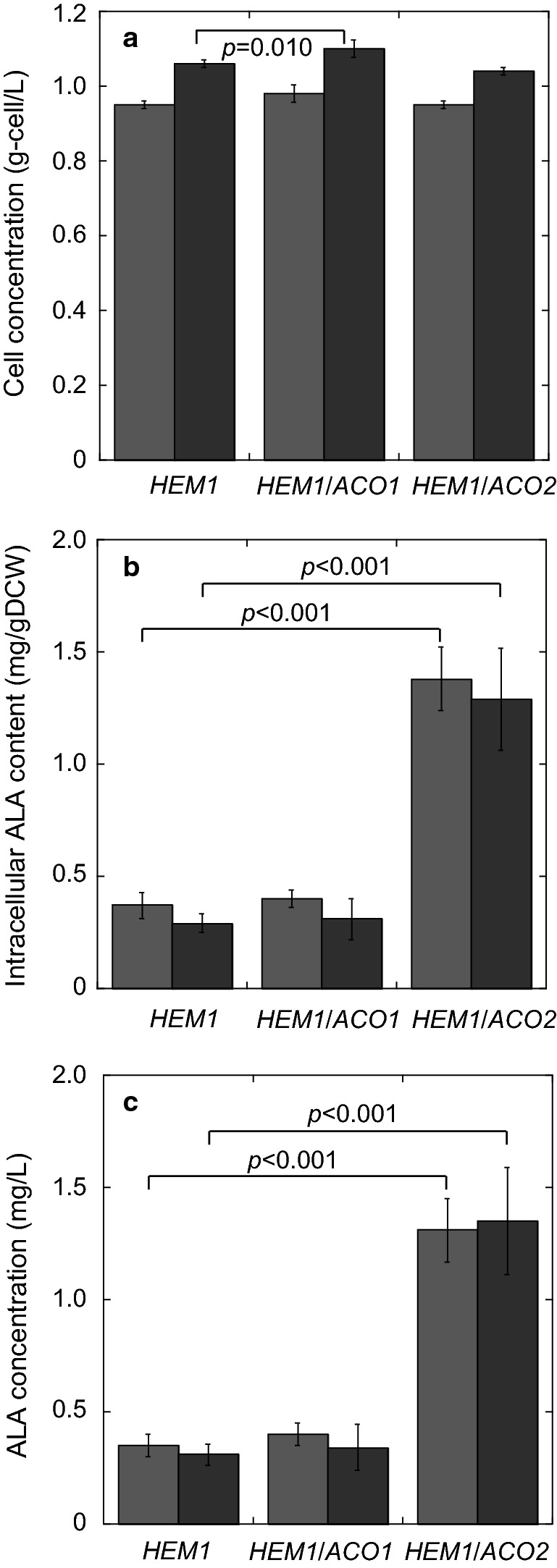
ALA production by combinatorial mutant strains affecting both ALA synthesis and citrate and isocitrate metabolism with 5 mM glycine added to the cultures. **a** Cell concentration (g-cell/L); **b** intracellular ALA content (mg/g DCW); **c** volumetric ALA concentration (mg/L). Gray and black bars represent values after cultivation for 24 and 48 h, respectively. The values are presented as means, with error bars showing SD (*n *= 3). *p*-values are represented when they show statistically significant differences among the engineered strains by Student’s *t*-test (*p* < 0.05, analysis of variance)

Next, to investigate the rate-limiting step of ALA biosynthesis in *HEM1*-overexpressing *S. cerevisiae*, we developed a method to measure multiple metabolites together with ALA. The conventional method of evaluating ALA biosynthesis using HPLC is not suitable for the measurement of amounts of multiple metabolites and ALA in *S. cerevisiae* at the same time. Previously, we developed a method to measure multiple metabolites using GC–MS [8]. Thus, in this study, we applied this GC–MS method to measure ALA content, and succeeded. After measuring the main metabolites and ALA production together, we found that the amount of citrate and isocitrate decreased with the increase in ALA production on addition of glycine to the *HEM1*-overexpressing strain (Table 1). As shown in Fig. 4, overexpression of *ACO2* enhanced ALA production. Overexpression of *ACO1* had less influence on ALA production, although it increased cell growth (Fig. 2a). These results indicate that isocitrate is a rate-limiting metabolite for ALA biosynthesis. Although both Aco1 and Aco2 affect the ALA production in *S. cerevisiae*, Aco2, rather than Aco1, catalyzes its biosynthesis when isocitrate is strictly limited; this occurs by enhancement of carbon flow to supply succinyl-CoA for ALA biosynthesis. The intracellular contents of some other metabolites were also changed by glycine addition to the *HEM1*-overexpressing strain (Additional file 1: Table S1). For example, the observed decrease in succinate would be caused because glycine addition stimulated Hem1 to convert succinyl-CoA into ALA (Fig. 1). In the future, a wider range of metabolic analyses would allow further prediction of the rate-limiting steps of ALA production in *HEM1*/*ACO2*-overexpressing *S. cerevisiae*, and these data would allow further improvement of ALA production.

## Methods

### Strains, plasmids, and media

NovaBlue (Novagen, Madison, WI, USA) was used as the *Escherichia coli* host strain for recombinant DNA manipulation. *S. cerevisiae* YPH499 (ABC1193) (*MATa ura3*-*52 lys2*-*801 ade2*-*101 trp1*-*Δ63 his3*-*Δ200 leu2*-*Δ1*) was used as the parental strain for metabolic engineering.

*E. coli* transformants were grown in Luria–Bertani medium (10 g/L tryptone, 5 g/L yeast extract, and 5 g/L sodium chloride) supplemented with 100 µg/mL ampicillin. Yeast transformants were cultured in Sabouraud dextrose agar (SDA; 6.7 g/L yeast nitrogen base without amino acids, 20 g/L glucose, and appropriate concentrations of amino acids). All chemicals were purchased from Nacalai (Kyoto, Japan), or Wako (Osaka, Japan).

### Plasmid construction and yeast transformation

The *HEM1* gene was amplified by PCR from *S. cerevisiae* YPH499 genomic DNA. The forward and reverse primers used for this amplification were: 5′-GGCCGCTAGCATGCAACGCTCCATTTTTGC-3′ (*Nhe*I site is underlined) and 5′-GGCCGGATCCTTACTGCTTGATACCACTAGAAAC-3′ (*Bam*HI site is underlined). The amplified fragment was digested with *Nhe*I and *Bam*HI, respectively, and inserted into the *Nhe*I/*Bam*HI sites of pGK405 to construct pGK405-*HEM1*. *ACO1* and *ACO2* were also amplified by PCR from *S. cerevisiae* YPH499 genomic DNA. The forward and reverse primers used for amplification of the *ACO1* gene were: 5′-GGCCGCTAGCATGCTGTCTGCACGTTCTGCC-3′ (*Nhe*I site is underlined) and 5′-GGCCGTCGACTTATTTCTTCTCATCGGCC-3′ (*Sal*I site is underlined). The amplified fragment of the *ACO1* gene was digested with *Nhe*I/*Sal*I and inserted into the *Nhe*I/*Sal*I sites of pGK406 to construct pGK406-*ACO1*. The forward and reverse primers used for amplification of the *ACO2* gene were: 5′-GGCCGCTAGCATGCTATCTTCAGCTAATAGG-3′ (*Nhe*I site is underlined) and 5′-GGAACCCGGGTTATTCGTTTCTTCGTATATTACC-3′ (*Xma*I site is underlined). The amplified fragment of the *ACO2* gene was digested with *Nhe*I/*Xma*I and inserted into the *Nhe*I/*Xma*I sites of pGK406 to construct pGK406-*ACO2*.

The *S. cerevisiae* YPH499 host strain was transformed with each plasmid (pGK405-*HEM1*, pGK406-*ACO1*, and pGK406-*ACO2*) digested by *Eco*RV. Transformants were selected by culture on SDA, and the insertions of the *HEM1*, *ACO1*, and *ACO2* genes were confirmed by PCR of genomic DNA using appropriate primers.

### Reverse transcription quantitative PCR

The relative mRNA levels of *ACO1* and *ACO2* were measured by RT-qPCR. Total RNA was extracted from yeast transformants by using NucleoSpin RNA Plus (Macherey–Nagel GmbH & Co., Düren, Germany). cDNA was synthesized using a PrimeScript™ RT Reagent Kit (Takara Bio Inc., Shiga, Japan), according to the manufacturer’s instructions. RT-qPCR was performed using a LightCycler^®^ Nano system and FastStart Essential DNA Green Master (Roche Diagnostics, Mannheim, Germany). Expression levels of target transcripts (*ACO1* and *ACO2*) were normalized to the *ACT1* mRNA level for each strain. RT-qPCR for *ACT1* and *ACO1* was performed using primer sets described in previous study [14], and the primers for *ACO2* (forward primer, 5′-AGGCTTATGACCTTGACGGAAC-3′; reverse primer, 5′-TCTTGCGGAACCTTCACCATAG-3′) were designed using the Primer3 plus program (https://primer3plus.com/).

### ALA fermentation using *S. cerevisiae* mutant strains

*Saccharomyces cerevisiae* mutant strains were cultivated in 5 mL yeast extract-peptone-dextrose (10 g/L yeast extract, 20 g/L Bacto Peptone and 20 g/L glucose) liquid medium at 30 °C with agitation at 200 rpm overnight. Each culture was inoculated into 50 mL Synthetic Defined liquid medium (6.7 g/L yeast nitrogen base without amino acids and 20 g/L glucose) in a baffled Erlenmeyer flask to an initial optical density at 600 nm (OD_600_) of 0.06. Cultures were then grown at 30 °C with agitation at 120 rpm for 24–48 h.

### Sample preparation for GC–MS analysis

For analysis of intracellular ALA content in *S. cerevisiae*, 15 mL of culture were collected after 24 and 48 h of cultivation. To quantify ALA, the culture containing yeast cells was centrifuged at 3000×*g* at − 20 °C for 5 min. Immediately, after centrifugation, cells were treated with a cold methanol method for quenching metabolism in yeast cells [15]—5 mL of precooled 60% (v/v) methanol solution at − 50 °C was added to the cell pellet and mixed quickly by inversion. After centrifugation at 3000×*g* at − 20 °C for 5 min, the cell pellet was frozen in liquid nitrogen and dried under vacuum. Metabolites were extracted from lyophilized cells using the previously described chloroform–methanol–water method [16]. The water phase of the extract solution (300 μL) was dried under vacuum and stored at − 80 °C until GC–MS analysis.

### GC–MS analysis

Dried extracts were derivatized using a method previously described [8, 17]. A fused silica capillary column (CP-Sil 8 CB low bleed, 30 m × 0.25 mm i.d., Film Thickness 0.25 µm; Varian Inc., Palo Alto, CA) was used for separation of the metabolites. A Shimadzu GCMS-QP-2010 system (Kyoto, Japan) was used for the detection of ALA and other metabolites. GC–MS analysis was performed using a modified method previously described [8]. In particular, *m/z* 174 was added to the ion monitoring channels for detection of ALA. A calibration curve was obtained from the ratio of ion peak areas of ALA and adipic acid as an internal standard detected at *m/z* 111.

## Supplementary information

**Additional file 1. Table S1.** Effect of glycine addition on intracellular metabolites in *HEM1*-overexpressing *S. cerevisiae.* **Figure S1.** Relative mRNA levels in HEM1-, HEM1/ACO1- and HEM1/ACO2-overexpressing strains.
