# Supplementary material for: 5-Aminolevulinic acid fermentation using engineered Saccharomyces cerevisiae
Source: Microb Cell Fact. 2019 Nov 7;18:194. doi: 10.1186/s12934-019-1242-6 (PMC6839092; doi:10.1186/s12934-019-1242-6)
Supplement: Supplementary file 1 — Additional file 1. Table S1. Effect of glycine addition on intracellular metabolites in HEM1-overexpressing S. cerevisiae. Figure S1. Relative mRNA levels in HEM1-, HEM1/ACO1- and HEM1/ACO2-overexpressing strains. [file 12934_2019_1242_MOESM1_ESM.doc]

**Table S1 Effect of glycine addition on intracellular metabolites in *HEM1*-overexpressing *S. cerevisiae***

| **Metabolite** | **2.5 mM**  **glycine** | **5.0 mM**  **glycine** |
| --- | --- | --- |
| Alanine | 12.99 | 13.51 |
| Aspartate | 5.85 | 4.06 |
| Fumarate | 0.40 | 0.99 |
| Glucose-6-phospahte | 1.27 | 2.10 |
| GABA | 1.20 | 0.88 |
| Glutamate | 1.85 | 0.58 |
| Glucose | 0.71 | 0.87 |
| Isoleucine | 2.46 | 1.90 |
| Lactate | 2.10 | 0.60 |
| Leucine | 1.79 | 0.79 |
| Mannitol | 0.71 | 0.85 |
| Mannose | 0.71 | 0.87 |
| Methionine | 1.32 | 1.19 |
| Phenyl alanine | 2.78 | 4.09 |
| Phosphorite | 0.84 | 0.89 |
| Proline | 0.54 | 0.21 |
| Pyruvate | 0.31 | 0.31 |
| Shikimate | 1.88 | 1.83 |
| Sorbitol | 2.00 | 1.11 |
| Succinate | 0.72 | 0.13 |
| Trehalose | 0.86 | 0.18 |
| Valine | 2.04 | 4.10 |

Samples were taken after cultivation for 24 h. Each value is the increase relative to the amount measured when glycine was not added to the culture. The culture conditions are described in “Methods.”


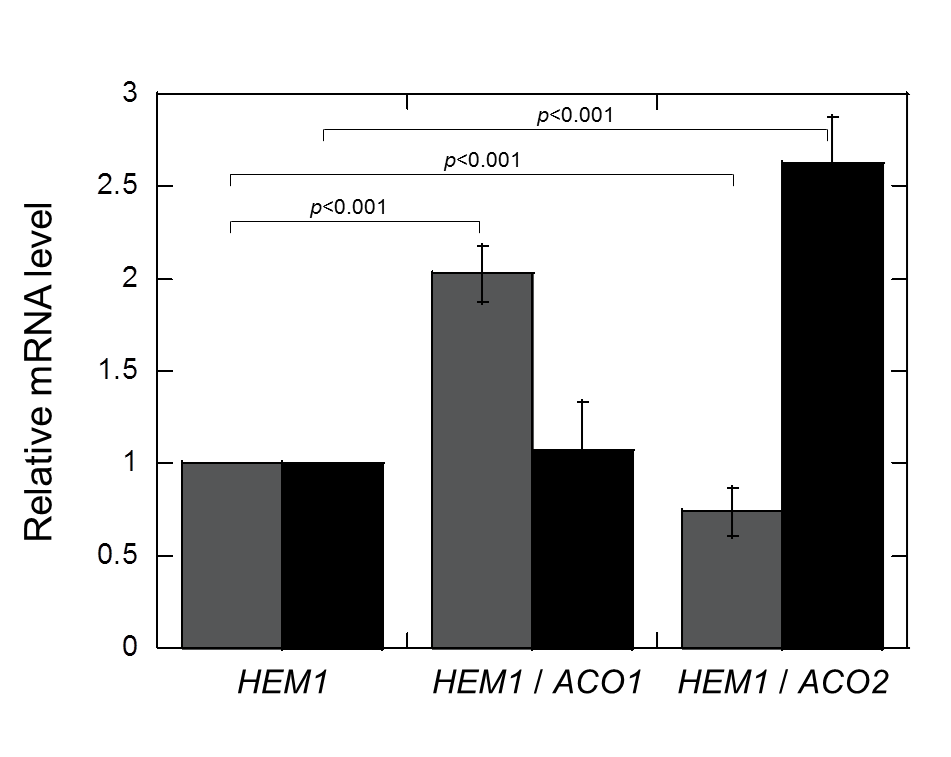
Figure S1. Relative mRNA levels in HEM1-, HEM1/ACO1- and HEM1/ACO2-overexpressing strains. Gray and black bars represent the relative mRNA level of ACO1 and ACO2, respectively, after normalization to the mRNA level of ACT1 as a reference gene. Samples were taken after cultivation for 24 h. The culture conditions and the method for reverse transcription quantitative PCR are described in “Methods.” The values are presented as means, with error bars showing SD (n = 3). P-values are represented when they show statistically significant differences among the engineered strains by Student’s t-test (p < 0.05, analysis of variance).
